# Supplementary material for: Huntingtin loss in hepatocytes is associated with altered metabolism, adhesion, and liver zonation
Source: Life Sci Alliance. 2023 Sep 8;6(11):e202302098. doi: 10.26508/lsa.202302098 (PMC10488683; doi:10.26508/lsa.202302098)
Supplement: Supplementary file 1 [file LSA-2023-02098_TableS1.docx]

| Parent A | Parent B | Expected | Observed | *p*-value |
| --- | --- | --- | --- | --- |
| *Htt^fl/+^;Alb^cre/+^* | *Htt^fl/+^;Alb^cre/+^* | 2.1875 | 2 | 0.89 |
| *Htt^fl/+^;Alb^cre/+^* | *Htt^fl/+^;Alb^cre/cre^* | 4.375 | 1 | 0.11 |
| *Htt^fl/fl^;Alb^cre/cre^* | *Htt^fl/+^;Alb^cre/cre^* | 34.5 | 36 | 0.97 |

Table S1. Mendelian ratios with expected and observed homozygote pups. Pearson’s chi-squared test p-values are reported for each pairing.
