# Supplementary material for: Huntingtin loss in hepatocytes is associated with altered metabolism, adhesion, and liver zonation
Source: Life Sci Alliance. 2023 Sep 8;6(11):e202302098. doi: 10.26508/lsa.202302098 (PMC10488683; doi:10.26508/lsa.202302098)
Supplement: Supplementary file 3 [file LSA-2023-02098_TableS2.docx]

| Tissue | *Htt^+/+^* | *Htt^LKO/LKO^* | *p-*value |
| --- | --- | --- | --- |
| Weight, g (SD) | 26.5 (2.4) | 24.4 (1.5) | **0.04** |
| Liver, mg (SD) | 1128.2 (179.3) | 1033.2 (195.8) | 0.28 |
| White adipose, mg (SD) | 719.4 (239) | 649 (150.8) | 0.5 |
| Brown adipose, mg (SD) | 50.1 (8.3) | 42.1 (6.8) | 0.09 |
| Spleen, mg (SD) | 84.2 (16.7) | 81.6 (16.7) | 0.73 |
| Brain, mg (SD) | 495.5 (12.2) | 496.2 (16.6) | 0.92 |

Table S2. Organ weights from 13-month mice. Significant t-test values are in bold.
