# Supplementary material for: Huntingtin loss in hepatocytes is associated with altered metabolism, adhesion, and liver zonation
Source: Life Sci Alliance. 2023 Sep 8;6(11):e202302098. doi: 10.26508/lsa.202302098 (PMC10488683; doi:10.26508/lsa.202302098)
Supplement: Supplementary file 4 [file LSA-2023-02098_TableS3.docx]

| Analyte (Unit) | *Htt ^+/+^* | *Htt ^LKO/LKO^* | *p*-value |
| --- | --- | --- | --- |
| Glucose (mg/dL) | 184.2 | 186.0 | 0.921 |
| BUN (mg/dL) | 17.7 | 22.8 | **0.002** |
| Creatinine (mg/dL) | 0.5 | 0.5 | 0.175 |
| BUN:creatinine ratio | 38.2 | 45.6 | **0.027** |
| Phosphorus (mg/dL) | 6.7 | 7.2 | 0.359 |
| Total Protein (g/dL) | 3.1 | 3.3 | 0.185 |
| Albumin (g/dl) | 1.5 | 1.6 | 0.465 |
| Globulin (g/dL) | 1.6 | 1.8 | 0.165 |
| Alb:Glob ratio | 1.0 | 0.9 | 0.363 |
| Bilirubin (mg/dL) | 0.1 | 0.2 | 0.123 |
| GGT (U/L) | 0.8 | 0.4 | 0.359 |
| ALT (U/L) | 105.5 | 114.6 | 0.886 |
| AST (U/L) | 214.8 | 229.5 | 0.838 |
| AST:ALT ratio | 3.1 | 2.5 | 0.440 |
| Cholesterol (mg/dL) | 41.2 | 55.6 | **0.003** |

Table S3. Analytes from clinical chemistry screen. Significant t-test comparisons (p < 0.05) are in bold. Abbreviations: blood urea nitrogen (BUN), gamma-glutamyl transferase (GGT), alanine aminotransferase (ALT), aspartate aminotransferase (AST).
