# Supplementary material for: Huntingtin loss in hepatocytes is associated with altered metabolism, adhesion, and liver zonation
Source: Life Sci Alliance. 2023 Sep 8;6(11):e202302098. doi: 10.26508/lsa.202302098 (PMC10488683; doi:10.26508/lsa.202302098)
Supplement: Supplementary file 7 [file LSA-2023-02098_TableS4.docx]

| Measure | *Htt^+/+^* | *Htt^LKO/LKO^* |
| --- | --- | --- |
| Lipid accumulation | 0 | 0 |
| Hepatocyte swelling | 0 | 0 |
| Fibrosis | 0 | 0 |
| Necrosis | 0 | 0 |
| Immune cell infiltration | 1 | 1 |
| Total score | 1 | 1 |

Table S4. Median liver pathology scores. Scores: 0 = absent, 1 = mild, 2 = moderate, 3 = severe.
