# Supplementary material for: Huntingtin loss in hepatocytes is associated with altered metabolism, adhesion, and liver zonation
Source: Life Sci Alliance. 2023 Sep 8;6(11):e202302098. doi: 10.26508/lsa.202302098 (PMC10488683; doi:10.26508/lsa.202302098)
Supplement: Supplementary file 8 [file LSA-2023-02098_TableS5.docx]

| Analyte (Unit) | LLOQ | *Htt^+/+^* | *Htt^+/+^* | *Htt^LKO/LKO^* | *Htt^LKO/LKO^* | *p*-value |
| --- | --- | --- | --- | --- | --- | --- |
|  |  | 6 mo | 13 mo | 6 mo | 13 mo |  |
| CCL6 (ng/mL) | 0.039 | 5.91 | 7.11 | 10.24 | 8.11 | **0.029** |
| CRP (ug/mL) | 0.33 | 3.19 | 4.02 | 4.03 | 4.26 | 0.129 |
| Eotaxin (pg/mL) | 9.4 | 4458.33 | 3526.67 | 4048.89 | 2917.78 | **0.021** |
| GCP-2 (ng/mL) | 0.35 | 1.15 | 0.78 | 1.31 | 1.06 | 0.177 |
| GM-CSF (pg/mL) | 1.2 | 8.24 | 2.59 | 11.51 | 1.62 | 0.462 |
| IL-12p40 (ng/mL) | 0.85 | 18.28 | 16.88 | 16.74 | 17.28 | 0.818 |
| IL-17A (pg/mL) | 12 | 74.6 | 27.32 | 117.02 | 23.26 | 0.268 |
| IL-1α (pg/mL) | 50 | 38.47 | 24.48 | 61.36 | 28.29 | 0.241 |
| IL-28 (pg/mL) | 6 | 71.7 | 10.23 | 86.12 | 9.99 | 0.529 |
| IL-5 (pg/mL) | 47 | 45.52 | 57.08 | 53.8 | 50.68 | 0.68 |
| IP-10 (pg/mL) | 23 | 58.45 | 52.82 | 63.44 | 44.58 | 0.15 |
| KC/GRO (pg/mL) | 6.7 | 89.62 | 67.92 | 61.16 | 63.69 | 0.096 |
| MCP-1 (pg/mL) | 19 | 28.37 | 23.53 | 29.36 | 44.7 | 0.115 |
| M-CSF-1 (ng/mL) | 0.06 | 0.61 | 0.52 | 0.52 | 0.47 | 0.248 |
| MDC (pg/mL) | 1.4 | 316.33 | 237.33 | 370.89 | 235.11 | 0.573 |
| MIP-1 β (pg/mL) | 0.88 | 10.83 | 11.98 | 11.01 | 10.84 | 0.599 |
| MIP-1γ (ng/mL) | 0.17 | 13.13 | 12.87 | 13.79 | 14.46 | 0.141 |
| MMP-9 (ng/mL) | 1.2 | 4.64 | 4.92 | 4.15 | 10.76 | 0.373 |
| SAP (ug/mL) | 2.6 | 65.23 | 90.42 | 80.39 | 95.91 | 0.132 |
| SCF (pg/mL) | 62 | 140.48 | 118.62 | 168.78 | 99.06 | 0.997 |
| TIMP-1 (ng/mL) | 0.62 | 1 | 0.67 | 1.25 | 1.05 | **0.026** |
| TNFα (pg/mL) | 14 | 134.92 | 23.53 | 214.67 | 24.1 | 0.148 |
| TSLP (pg/mL) | 5.3 | 20.35 | 6.39 | 29.18 | 4.97 | 0.445 |
| VCAM-1 (ng/mL) | 2.2 | 593.67 | 715.83 | 603.78 | 846.89 | 0.053 |
| VEGF-A (pg/mL) | 4.2 | 11.31 | 11.27 | 13 | 9.28 | 0.851 |

Table S5. Analytes from rodent MAP4.0 (Ampersand Biosciences). Significant genotype ANOVA comparison (*p* < 0.05) are in bold. Abbreviations: Chemokine (C-C motif) ligand (CCL), C-reactive protein (CRP), Granulocyte Chemotactic Protein (GCP), Granulocyte-Macrophage Colony-Stimulating Factor (GM-CSF), interleukin (IL), interferon gamma-induced protein (IP), Growth-Regulated Protein alpha (KC/GRO), monocyte chemoattractant protein (MCP), macrophage colony-stimulating factor (M-CSF), macrophage-derived chemokine (MDC), macrophage inflammatory proteins (MIP), Matrix metallopeptidase (MMP), Serum Amyloid P component (SAP), Stem Cell Factor (SCF), tissue inhibitor of metalloproteinase (TIMP), Tumor Necrosis Factor (TNF), Thymic stromal lymphopoietin (TSLP), Vascular cell adhesion protein (VCAM), vascular endothelial growth factor-A (VEGF-A). Inflammation markers measured below lower limit of quantitation (LLoQ with LLoQ listed: IFNβ (112 pg/mL), IFNγ (1.1 pg/mL), IL-10 (5.7 pg/mL), IL-12p70 (21 pg/mL), IL-18 (208 pg/mL), IL-1β (3 pg/mL), IL-2 (11 pg/mL), IL-23 (35 pg/mL), IL-27 (28 pg/mL), IL-4 (2.8 pg/mL), IL-6 (3.9 pg/mL), IL-9 (132 pg/mL), Insulin (40 mUI/ml), MIP-1α (221 pg/mL), PAI-1 (0.26 ng/mL), RAGE (0.42 ng/mL), TPO (0.046 ng/mL).
