# Supplementary material for: Huntingtin loss in hepatocytes is associated with altered metabolism, adhesion, and liver zonation
Source: Life Sci Alliance. 2023 Sep 8;6(11):e202302098. doi: 10.26508/lsa.202302098 (PMC10488683; doi:10.26508/lsa.202302098)
Supplement: Supplementary file 11 [file LSA-2023-02098_TableS6.docx]

| Experiment | Age | N/group | Sex | Statistical tests |
| --- | --- | --- | --- | --- |
| Bodyweight | 3-12 months | 11 WT, 20 LKO | Female | Repeated measure ANOVA |
| Medelian ratio | 1 month | See Table S1 | Mixed | Pearson's chi-squared |
| HTT protein blots | 12 month | 4 | Mixed | T-test, bonferroni adjustment |
| Organ weights | 13 months | See Table S2 | Female | T-test |
| Liver pathology scoring | 6 months | 5 WT, 11 LKO | Female | N/A |
| Plasma chemistry | 6 months | 11 | Female | T-test |
| Bile acid measurement | 2 months | 163 WT, 34 LKO | Female | T-test |
| GTT | 6, 13 months | 10 | Female | T-test |
| ITT | 6, 13 months | 10 | Female | T-test |
| PTT | 6, 13 months | 10 | Female | T-test |
| Liver blistering | 3-9 months | variable | Mixed | N/A |
| RNAseq - DEGs | 9 months | 6 | Female | Moderated T-test |
| EnrichR Pathways | 9 months | 6 | Female | Odds ratio |
| IHC - CYP1A2 | 6-months | 6 | Male | T-test |
| IHC - CYP2E1 | 6-months | 6 | Male | T-test |
| IHC - CDH1 | 6-months | 6 | Male | T-test |
| IHC - CDH2 | 6-months | 3 | Male | T-test |
| APAP challenge | 6-months | 9 WT, 7 LKO | Male | T-test |

Table S6. Summary of age, N, sex, and statistical test for each experiment.
